# CRISPR-based Transcriptional Regulation: Technologies, Applications, and Future Directions

**DOI:** 10.3390/dna5040057

**Published:** 2025-12-01

**Authors:** Mira A. Srinivasa, Mario Escobar

**Affiliations:** 1 Department of Bioengineering, Rice University; Houston, TX 77005, USA; 2 Michael E. DeBakey Department of Surgery, Baylor College of Medicine; Houston, TX 77030, USA

**Keywords:** CRISPR-Cas Systems, Genetic Engineering, DNA, Epigenetics

## Abstract

CRISPR-based transcriptional regulation technologies, including CRISPR activation (CRISPRa) and CRISPR interference (CRISPRi), offer precise and programmable control over gene expression, representing a major advance in gene and epigenetic therapy. CRISPRa uses nuclease-inactive Cas proteins fused to transcriptional activators to upregulate target genes, while CRISPRi employs repressor domains for gene silencing. Preclinical studies have demonstrated the efficacy of CRISPRa/i in models of metabolic, neurological, muscular, and oncological diseases. Notably, CRISPRi-based therapies have entered clinical trials for conditions like hepatitis B and muscular dystrophy, showing encouraging safety and efficacy profiles. Despite ongoing challenges related to delivery efficiency, immunogenicity, and off-target activity, innovations in protein engineering and guide RNA design are rapidly enhancing the precision and safety of these technologies. Overall, CRISPRa and CRISPRi are poised to transform the treatment of genetic and epigenetic disorders, with continued optimization expected to accelerate their clinical adoption and broaden their therapeutic impact.

## 1. Introduction

For over ten years, CRISPR (Clustered Regularly Interspaced Short Palindromic Repeats) technologies have driven the gene editing landscape [1–3]. CRISPR/CRISPR-associated (Cas) nuclease systems were originally derived from bacterial defense systems evolutionarily designed to recognize and silence foreign DNA or RNA sequences [4–6]. Yet through extensive engineering, the field has repurposed these endogenous systems into versatile molecular tools, particularly the Class II Cas9 proteins.

In their native form, Cas9 systems consist of a Cas9 DNA endonuclease loaded with two RNA molecules: a CRISPR RNA (crRNA), which recognizes and binds to a target sequence, and a transactivating crRNA (tracrRNA), which facilitates crRNA processing and Cas9-crRNA complexing [1,4]. However, most current applications of the Cas9 systems utilize a single guide RNA (sgRNA or gRNA), a synthetic RNA that combines the crRNA and tracrRNA, streamlining Cas9 usage [1]. Following RNA loading into the Cas9 protein, the complex will scan through the genome, stopping at 2–5 bp protospacer adjacent motif (PAM) sites to locate a 17–22 bp region of DNA complementary to the protospacer. After binding to the target site, the Cas9 induces a double-stranded break (DSB) with blunt ends, located 3 bp upstream of the PAM [7,8]. Taking advantage of DNA repair mechanisms, this break enables the deletion of endogenous genomic loci or insertion of exogenous genetic sequences [7,9–11]. Details regarding these mechanisms and applications have been extensively reviewed in the literature.

In addition to the canonical Cas9, there are additional Class II Cas proteins with distinct functionalities available in the molecular toolbox, particularly Cas12a (previously Cpf1), and Cas13 (previously C2c2) [12,13]. While Cas9 requires an individual mature sgRNA, Cas12a is able to process multiple sgRNAs from a single transcript, thanks to its intrinsic RNase activity, allowing for multiple sgRNAs to be used simultaneously (multiplexing). Additionally, Cas12a DNA cleavage generates sticky ends upon inducing the DSB, decreasing homology dependence [12]. In contrast, Cas13 targets and cleaves RNA rather than DNA, giving rise to mRNA-targeting technologies with minimal genomic alterations, and even isoform-specific targeting [14,15]. The diversity of Cas proteins is further expanded when taking into consideration distinct protein orthologs from various microbial species. While the conventional Cas9 is derived from *Streptococcus pyogenes* (SpCas9), Cas9 variants from *Staphylococcus aureus*, *Streptococcus thermophilus*, and *Campylobacter jejuni* have also been explored for biomedical applications due to their PAM targeting requirements, target specificity, and Cas size [4,16–19]. Beyond repurposed native Cas systems, the available Cas systems have also increased through synthetic “high-fidelity” mutants engineered to enhance Cas specificity and editing efficiency [20,21]. This expanding toolkit has highlighted the need for systematic standardization and direct comparison between Cas systems, an ongoing and pressing need [21–24].

The precision of CRISPR-mediated gene editing has catalyzed its exploration as a therapeutic strategy for genetic diseases. In December 2023, Casgevy, an *ex vivo* CRISPR/Cas9 therapy for sickle cell disease, became the first FDA-approved CRISPR-based therapeutic [25]. Furthermore, numerous phase II–III clinical trials are underway for indications including blood disorders, leukemia, immunodeficiencies, muscular dystrophies, Huntington’s disease, ALS, and metabolic conditions [26–32]. Another major CRISPR-based clinical advance is base editing, in which either a nuclease-deactivated Cas protein (dCas) or a nickase Cas (nCas) is fused to a cytidine or adenine deaminase, enabling the conversion of cytosine to thymine (cytosine base editors, CBEs) or adenine to guanine (adenine base editors, ABEs) without inducing a DSB [33]. Notably, a patient-specific ABE was published in May 2025 for an emergency case of neonatal-onset carbamoyl-phosphate synthetase 1 (CPS1) deficiency, with no evidence of significant adverse effects [34]. In addition to its application in CPS1 deficiency, base editing is being actively investigated in other applications across numerous clinical trials [35–38].

The precise targetability of CRISPR systems has been harnessed not only for genome editing but also for transcriptional regulation [39–42]. The binding of Cas proteins to DNA is controlled by both the recognition of the PAM and the sequence complementarity between the sgRNA and the genomic target. Thus, inactivation of the Cas nuclease domains still maintains the protein targeting functionality and generates a non-cleaving programmable DNA-binding platform [39]. By fusing transcriptional activators to this nuclease-inactive Cas (dCas), researchers developed the CRISPR activation (CRISPRa) tool for precise and robust upregulation of target gene expression [40–42]. Conversely, fusion of transcriptional repressors to the dCas protein gives rise to the CRISPR interference (CRISPRi) tools [39,40,43]. In this review, we provide a comprehensive update on the state of diverse CRISPRa and CRISPRi technologies in translational applications.

## 2. CRISPRa

### 2.1. Transcriptional Activation: Benefits and Limitations

Targetable transcriptional activation holds significant promise for treating human diseases driven by transcriptional repression. For example, reactivation of tumor suppressor genes is a promising strategy in cancer treatment; restoration of immune response gene expression may counteract the immunosuppressive effects of SARS-CoV-2 infection; and enhancing the transcription of synaptic transmission genes could alleviate deficits observed in Alzheimer’s disease [44–46]. Traditionally, transcriptional deficiencies have been addressed through direct protein replacement. For example, enzyme replacement therapies for Gaucher disease, a pathology characterized by insufficient levels of the lysosomal enzyme β-glucocerebrosidase, began gaining FDA approval in the early 1990s [47]. However, direct protein delivery is limited by short protein half-lives and inefficient cellular uptake, necessitating frequent and often expensive infusions. Furthermore, the administration of exogenous proteins can provoke adverse immune responses [47].

Alternatively, gene supplementation via cDNA delivery has also been developed [48]. Skysona is an FDA-approved cDNA-based therapy for cerebral adrenoleukodystrophy (CALD). In this treatment, hematopoietic stem and progenitor cells (HSPCs) are harvested from the patient and transduced with a lentiviral vector encoding the cDNA for ATP-binding cassette domain 1 (ABCD1), the protein deficient in CALD [49,50]. Despite its therapeutic potential, cDNA addition can result in supraphysiological gene expression, which may lead to unintended and potentially harmful effects [51].

CRISPR activation (CRISPRa) systems offer a more refined and adaptable strategy, enabling precise upregulation of endogenous gene expression to physiologically relevant levels. This tunability makes CRISPRa a compelling platform for therapeutic gene activation. The following subsections will review the current landscape of Cas-activator systems and discuss their translational progress toward clinical application.

### 2.2. CRISPRa Formulations

CRISPRa systems display considerable variability in their capacity to upregulate gene expression. Early CRISPRa platforms typically utilized a dCas9 protein fused to the synthetic transcriptional activator VP64, itself composed of four tandem repeats of the herpes simplex virus activator VP16 [40–42,52]. When paired with an sgRNA targeting a specific gene, this fusion can induce robust and precise increases in gene expression, up to a thousand-fold, depending on the gene target and cell line [42]. An underscored limitation of the systems, however, is that such high levels of activation generally require the delivery of multiple sgRNAs, as single sgRNAs typically produce more modest activation of approximately 2- to 10-fold [40,42,53]. Beyond VP64, other transcriptional activators such as p65 and Rta have been explored, though neither dCas9-p65 nor dCas9-Rta consistently matched the activation efficiency observed with dCas9-VP64 [54].

Chromatin-modifying domains have also demonstrated high efficacy in mammalian cells, directly altering chromatin structure to promote gene activation. The dCas9-p300 (histone acetyltransferase) and PRDM9-dCas9 (histone methyltransferase) fusions described by Hilton et al and Cano-Rodriguez et al, respectively, activate genes via histone alterations [55–57]. This approach demonstrates strong potential in gene activation, as dCas9-p300 increased expression nearly 7 times more than dCas9-VP64 at certain genes [55]. Additionally, PRDM9-dCas9 was shown to successfully and stably induce re-expression of previously silenced genes in regions with low levels of DNA methylation [57]. Chromatin-modifying domains, then, are heavily influenced by the surrounding chromatin environment, emphasizing the need for clinical validation in the tissue of interest prior to clinical implementation. Beyond histone modifications, the Tet dioxygenases, including Tet1 and Tet3, can induce expression at silenced genes via DNA demethylation [58,59]. dCas9-Tet3 successfully reduced target gene methylation, increased hydroxymethylation, and reduced kidney fibrosis *in vivo*, demonstrating its therapeutic potential at hypermethylated genes [59]. Likewise, dCas9-Tet1 achieved significant decreases in DNA methylation and increases in expression at a previously silenced reporter gene *in vivo* [58]. These domains suggest that the diverse mechanisms of CRISPRa systems offer an array of options to control the extent, duration, and tissue specificity of target gene expression.

Further improvements in activation efficiency have been achieved by increasing the number or diversity of activator domains fused to dCas9. For example, a VP64-dCas9-VP64 construct significantly increased the expression of *Myod1*/*MYOD1* by approximately 40–60 -fold and 4-fold in murine and human cells, respectively, when using a single sgRNA. In contrast, single dCas9-VP64 fusions did not yield significant changes utilizing the same single sgRNA [60]. Similarly, a simultaneous VP64-p65-Rta (VPR) fusion yielded significant increases in expression over any monomeric or dimeric fusions, supporting the utility of multiple transcription factors in CRISPRa platforms [54]. Protein recruitment systems, such as SunTag, have also been employed to recruit up to 10 VP64 proteins to a single dCas9, increasing target gene expression significantly more than dCas9-VP64, up to 50-fold, with notable effects on cell function [52,61]. The CRISPR synergistic activator mediator (SAM) involves a dCas9-VP64 core and a fusion of the bacterial coat protein MS2 with p65 and heat shock protein 1 (HSF-1), which is bound to the sgRNA. CRISPR SAM achieved significant improvement over dCas9-VP64 for multiple gene targets [62].

These domains have also been tested beyond dCas9. dCas12a’s ability to process its own crRNAs allows multiple gRNAs to be derived from a single transcript, and hence, facilitates multiplexed CRISPR activation. When fused to VPR, p65, HSF-1, or even protein-tagging systems, it has successfully activated up to 10 genes simultaneously [63–65]. Such multiplexing, however, naturally bears a greater risk of off-target activation and decreased specificity [66].

Thanks to its RNA-targeting ability, dCas13-based systems have also been used to increase gene activity through post-transcriptional modulation. Unlike DNA-targeting systems, dCas13-based activation increases protein production without directly affecting the genome, allowing for more short-term regulation [67,68]. CRISPRa at the RNA level also allows for more direct control of protein levels, circumventing potential nonlinearities between the amount of expression and protein quantity [69]. In these technologies, domains such as PABPC1, N-acetyltransferase 10 (NAT10), and m^6^A demethylase AlkB homolog 5 (ALKBH5), which increase mRNA stability and translation, are fused directly to the dCas13 [70–72]. It is important to note, though, that dCas13-ALKBH5 yielded variable effects at different transcript targets [70]. Characterizing the effects at the precise mRNA of interest would be crucial to clinical implementation. Additionally lncRNA-derived activating elements, such as SINEB2, have been coupled to the sgRNA itself. This latter method of RNA-driven activation is promising for delivery, as the small size of the unfused enzyme alone enables delivery within a single viral particle [67]. While such dCas13 tools allow for highly-specific RNA-level regulation, with applications in RNA diagnostics and therapeutics, they have the potential to be further engineered and optimized. Further potential avenues involve base-editing fusions such as dCas13b-ADAR2, composed of an adenosine deaminase acting on RNA (ADAR) enzyme, that employ deactivated Cas13 to correct precise disease-causing mutations [73].

Table 1 provides a list of transcriptional activators and novel enhancements that have been employed in CRISPRa systems.

### 2.3. CRISPRa Therapeutic Applications

This large variety and success of CRISPRa platforms and the utility of precise gene upregulation in human diseases emphasize the clinical value of CRISPRa for diverse organ systems. Current *in vivo* applications are discussed below.

#### 2.3.1. Obesity

Obesity is an urgent, growing health issue compounded by many common comorbidities, including Type II diabetes and heart disease [76]. It is characterized by excessive adipose tissue mass [77]. Mammals contain both brown adipose tissue (BAT) and white adipose tissue (WAT), which expend and store excess energy, respectively [78]. Several groups have thus employed CRISPRa to activate processes characteristic of BAT to increase energy expenditure in adipose tissue as a potential treatment for obesity [76,78].

Zhu et al used dCas9-VP64 to precisely activate the fibroblast growth factor 21 (*Fgf21*) and fibronectin type III domain-containing protein 5 (*Fndc5*) [76]. These two proteins, secreted by muscle cells, are crucial for driving BAT-like energy dissipation in WAT, but tend to be expressed at lower levels in obesity [76]. Increased *Fgf21* and *Fndc5* expression after CRISPRa treatment was first validated in mouse C2C12 myoblasts. Importantly, AAV delivery of dCas9-VP64 and sgRNA for the two target genes specifically increased transcript levels, reduced body weight, and lowered fat accumulation in mouse models of obesity [76].

Wang et al applied CRISPR-SAM to increase expression of uncoupling protein 1 (*UCP1*), a gene specifically expressed in BAT cells, in WAT cells. These reprogrammed WAT cells were then mixed with Matrigel prior to implantation into mouse models of obesity [78]. Treated mice exhibited higher levels of energy expenditure and heat production, improved glucose metabolism and insulin sensitivity, and slower rates of weight gain under high-fat diets [78]. This study extends the therapeutic potential of CRISPRa past direct delivery to regenerative or therapeutic cell lines.

Matharu et al also explored CRISPRa for obesity from a haploinsufficiency approach [79]. Haploinsufficiency, in which only one copy of a gene is functional, of single-minded family bHLH transcription factor 1 (Sim1) or melanocortin 4 receptor (Mc4r), are directly linked to excessive hunger and obesity. dCas9-VP64 and sgRNAs targeting the *Sim1* promoter and a distant enhancer significantly increased gene expression *in vitro* in mouse neuroblastoma cells. Additionally, delivery of the CRISPRa system via rAAV significantly reduced weight in haploinsufficient Sim1^+/−^ mice with induced obesity [79]. These results were validated through observed increases in *Mcr4* expression and decreased weight gain after CRISPRa rAAV delivery in haploinsufficient Mcr4^+/−^ mice with induced obesity [79].

Comprehensively, these results demonstrate the clinical applicability of CRISPRa tools in metabolic disorders through diverse approaches targeting adipose tissue and endocrine glands.

#### 2.3.2. Neurological Disorders

CRISPRa has also been explored extensively in neurological disorders, including Parkinson’s, Alzheimer’s, and epilepsy, due to their extensive genetic risk factors.

Brain astrocytes have been shown to play an instrumental role in converting dopamine precursor, L-DOPA, to dopamine in Parkinson’s via the tyrosine hydroxylase (Th) enzyme [80]. Narváez-Pérez et al used a lentiviral vector to stably express dCas9-SAM and an sgRNA targeting *Th* in a primary rat astrocyte line. Treated cells displayed increased levels of Th and secreted dopamine into the surrounding media, unlike controls. Moreover, striatal implantation of these CRISPRa-treated cells into surgically-lesioned rat models of advanced Parkinson’s, 6-OHDA rats, yielded improved performance in behavioral and motor tests and increased dopamine metabolism compared to untreated rats [80]. These functional tests validate the role of CRISPRa-treated cells in clinically relevant applications, improving both physiological and symptomatic disease features. In another Parkinson’s application, Giehrl-Schwab et al co-injected SAM-containing and dCas9-VP64-containing AAVs into the dorsal striatum of 6-OHDA rats. sgRNA targeted the *Ascl1, Lmx1a*, and *Nr4a2* genes, which all code for transcription factors [81]. This treatment effectively reprogrammed striatal astrocytes into GABAergic neurons, which displayed action potentials characteristic of endogenous neurons, and functionally improved average motor speed and stride length. These motor improvements were more variable, though, lacking significant change in certain measures such as rotational capability [81]. Nevertheless, their study encourages *in vivo* delivery of robust CRISPRa systems for multiplexed gene activation and tissue reprogramming.

In Alzheimer’s disease, activation of α-secretase minimizes the development of pathological amyloid-β plaques by shifting away from amyloidogenic β- and γ-secretase pathways [82]. Increased expression of *Adam10*, a member of the α-secretase family, was previously linked to plaque reduction and improved memory and learning in mouse Alzheimer’s models, though therapeutic effects were achieved via crossbreeding [83]. Park et al injected an *Adam10*-targeting dCas9-SAM nanocomplex, constructed via amphiphilic peptide incubation, into the hippocampus of 5xFAD mice Alzheimer’s models [82]. Nanocomplex delivery significantly increased *Adam10* expression, decreased levels of Aβ42, a more amyloidogenic amyloid-β isoform, and ultimately reduced plaque formation. Importantly, measures of immunogenicity and toxicity, including cytokine secretion, microglial markers, and apoptosis indicators, did not change significantly. Additionally, nanocomplex-treated 5xFAD mice performed better in maze tests and exhibited improvements in long-term memory and fear response [82]. Future studies by Park and Kim used dCas9-VPR to activate *Mt1*, a melatonin receptor isoform that has been shown to increase anti-inflammatory neuroprotection, learning, memory, and plaque degradation [84]. Lentiviral injection of *Mt1* CRISPRa into 5xFAD hippocampi significantly decreased levels of Aβ42. Additionally, maze test performance, memory, and fear response improved upon *Mt1* CRISPRa treatment in the Alzheimer’s models [84].

Aberrant regulation of the ion channel K-Cl cotransporter isoform 2 (KCC2) has been shown to play a prominent role in epilepsy. Specifically, increasing *KCC2* expression may be especially important in mitigating drug resistance [85]. In a study by Shi et al, AAV-mediated delivery of dCas9-SAM and an sgRNA targeting murine *Kcc2* into the inferior hippocampus significantly reduced the severity and duration of generalized seizures [85]. Drug responsiveness was tested following CRISPRa delivery and intraperitoneal injection of diazepam, an epilepsy treatment that improves neurotransmitter signaling. Diazepam and CRISPRa co-treatment significantly decreased seizure severity and duration in comparison to controls or either treatment alone. CRISPRa co-treatment also enhanced the effectiveness of diazepam when administered 30–60 minutes post-seizure, by when it is typically ineffective. Results were further validated with valproate, another epilepsy drug [85]. Colosante et al targeted the potassium channel gene *Kcna1*. dCas9-VP64 and *Kcna1* sgRNA were injected via AAV into the primary visual cortex, significantly reducing the number of seizures per day from 7 to 21 days post-injection. A non-significant, but promising, reduction in seizure severity was also observed in an acute seizure model [86]. More extensive benefits were also observed, as *Kcna1* CRISPRa treatment improved cognitive performance and restored normal gene expression in over one-third of the dysregulated epileptic genome [86]. CRISPRa can thus yield widespread phenotypic improvements with a single delivery.

The use of CRISPRa in nervous system applications extends further to psychiatric conditions such as alcohol use disorder, which also involves epigenetic changes. The activity–regulated cytoskeleton-associated protein (*Arc*) gene is heavily involved in neuronal plasticity and is downregulated following early alcohol exposure. Bohnsack et al explored the use of a dCas9-p300 system with four *Arc*-targeted sgRNAs in adult rats given high levels of alcohol during adolescence, which can increase anxiety and alcohol intake in adulthood [87]. dCas9-p300 and the sgRNAs were injected via lentivirus into the central nucleus of the amygdala (CeA), a region of the brain linked to alcohol use and anxiety disorders. For rats previously exposed to high levels of alcohol, CRISPRa-treatment reduced both anxious behavior and alcohol intake levels to those characteristic of non-exposed rats. These behavioral results were corroborated by precise increases in *Arc* expression and restored histone acetylation, with no observed off-target effects [87]. Their study demonstrates the broad applicability of epigenetic therapies for more complex behavioral diseases.

#### 2.3.2. Musculoskeletal Disorders

Muscular dystrophy has been a common target for gene editing and traditional CRISPR/Cas9 technologies. It is more recently being explored through gene regulation, and notable epigenetic changes have been observed in Duchenne and Emery-Dreifuss muscular dystrophies [88]. CRISPRa-mediated therapy is thus a viable area of exploration. Utrophin (*UTRN*) is an autosomal analog of dystrophin, which is dysfunctional in Duchenne muscular dystrophy (DMD). *UTRN* activation can thus help mediate the lack of sufficient dystrophin protein, which causes muscle weakness and degeneration [88]. Additionally, small-molecule approaches for utrophin activation have been promising in clinical trials. Wu et al delivered a miniaturized CRISPRa platform, dCas9MINI-VPR, with sgRNA targeting *Utrn* via intramuscular AAV injection, using two distinct DMD mouse models. Utrophin mRNA and protein levels significantly increased after treatment, with precise localization to dystrophin-rich regions in WT controls [89]. Muscle tissue of treated mice also exhibited reduced inflammation, damage, and fibrosis up to six months post-treatment. Functionally, treatment improved muscle strength, grip strength, and locomotor abilities and mitigated heart failure, a common cause of death in DMD. Subsequent experiments in non-human primates also demonstrated significant utrophin upregulation in muscle tissue without notable side effects. Together, these results demonstrate the clinical significance and longitudinal benefit of CRISPRa therapies for DMD [89]. Importantly, in 2023, Lek et al attempted to activate the Dp427c dystrophin variant via rAAV delivery in a 27-year-old DMD patient. The dCas9-VP64 construct effectively increased *Dp427c* expression *in vitro* and *in vivo* in murine models; however, throughout the week following treatment, the patient developed several life-threatening multiple organ conditions, including cardiac failure and ARDS. However, the authors believe that the death was due to a toxic immune response from the high dose of rAAV, and the effects of CRISPRa could not be analyzed [90].

Merosin-deficient congenital muscular dystrophy type 1A (MDC1A) is another form of muscular dystrophy, caused by mutations in *LAMA2*, which encodes the α−2 subunit of the laminin 2 protein [91]. Activation of laminin-α1 (*LAMA1*) can help restore this lost function. However, the size of the *LAMA1* gene limits direct delivery via smaller viral vectors. Kemaladewi utilized a VP64-dCas9-VP64 complex with three sgRNA targeted to *Lama1* in a mouse model of MDC1A. AAV-based intramuscular injection significantly increased *Lama1* mRNA and protein expression in treated muscle. Additionally, improvements in fibrosis and muscle fiber morphology, as well as overall mobility, muscle force generation, and nervous system coordination were observed [91]. Liu et al validated the *in vivo* viability of CRISPRa-induced *Lama1* activation for MDC1A using a more severe, early lethality mouse model [92]. Significant increases in the expression of *Lama1* at the mRNA and protein levels were observed following subcutaneous AAV injection of CRISPR SAM and two *Lama1* sgRNA. Additionally, *Lama1* activation significantly increased median survival time, body weight, grip strength, and muscle pathology. While these measures remained significantly lower than WT mice, the results suggest epigenetic regulation can improve health even in severe diseases. An important consideration, however, remained AAV-induced toxicity at high doses [92]. As CRISPRa continues to demonstrate promising *in vivo* potential, optimizing dosage, delivery, and uptake will be crucial for future clinical applications.

## 3. CRISPRi

### 3.1. Transcriptional Repression: Benefits and Limitations

As opposed to CRISPRa, for diseases characterized by abnormal overexpression, precise transcriptional repression serves as a promising therapeutic target. Potential applications include angiogenic and anti-apoptotic genes in cancer, host genes that respond to viral and bacterial infection, inflammatory genes in autoimmune disorders, and plaque-forming genes in Alzheimer’s [93–95]. Protein inhibition can be achieved directly via small molecules and biologics. However, these therapies, which include monoclonal antibodies and synthetic drugs, are limited by factors such as short half-lives, low cellular permeability, and poor tissue distribution [96]. Numerous methods also exist to inhibit gene expression at the DNA and RNA levels. Anti-sense oligonucleotides (ASOs) are synthetic, single-stranded nucleic acids that bind to pre-mRNA or mRNA via complementary base pairing. Inhibition is achieved via splice interference, obstruction of ribosomal attachment and translation initiation, and/or transcript degradation [97]. Their relatively small size (~13–30 nts) enhances ASO delivery, but off-target localization in the body and degradation by cellular and systemic nucleases limits their function [97,98]. Thirteen ASO-based drugs are currently FDA- or EMA-approved [99]. However, because they work post-transcriptionally, repeated dosing is needed for continued inhibition. In contrast to ASOs, RNA interference (RNAi) starts with a double-stranded RNA. One strand then associates with the endonuclease Ago 2 to form the RNA-induced silencing complex (RISC), which binds and cleaves the target mRNA [98]. small interfering RNA (siRNA) refers to the double-stranded RNA molecule and has been explored extensively in clinical trials and five FDA-approved products [100]. Similar to ASOs, though, siRNAs are limited by off-target binding and systemic degradation, as well as immunogenic activation of Toll-like receptor 3 (TLR3) [94].

As an alternative to these protein and nucleic acid inhibitors, CRISPR interference (CRISPRi) functions similarly to its activating analog. dCas9 fused to a transcriptional repressor can target, bind, and decrease expression of specific genes at the transcriptional level. Previous comparisons have suggested that CRISPRi exhibits decreased rates of off-target binding compared to RNAi while maintaining similar silencing specificity [40,101]. In the following sections, we describe current CRISPRi systems and their therapeutic applications.

### 3.2. CRISPRi Formulations

In its simplest form, Qi et al showed that dCas9 and sgRNA alone could decrease expression at the target gene by sterically hindering RNA polymerase, roughly halving expression [39,40]. However, fusion of transcriptional repressors improves the efficiency of dCas9 alone via the recruitment of chromatin effectors [40]. The Krüppel-Associated Box (KRAB) family of proteins was among the first repressors to be used with dCas9 and achieved up to 15-fold repression in early experiments in HEK293 cells [40]. Thakore et al also demonstrated that dCas9-KRAB could be targeted to enhancers to decrease chromatin accessibility, induce histone methylation, and may even affect 3D chromatin interactions driven by loop contact. Their findings build on previous studies and expand the range of genomic sites from which repression can be achieved [102].

Additional repressor domains have been used alongside KRAB to further increase inhibitory efficiency. For example, methyl-CpG binding protein 2 (MeCP2) functions by regulating histone deacetylase (HDAC) interactions with DNA [103]. In early studies, dCas9-KRAB-MeCP2 improved gene inhibition compared to dCas9-KRAB alone at most target sites [104]. Additionally, several groups have explored KRAB family members beyond the canonical KRAB domain of the human *KOX1* gene. The KRAB domain from the *ZIM3* gene, for example, (dCas9-KRAB_ZIM3_), has been shown to outperform both dCas9-KRAB and dCas9-KRAB-MeCP2 [105]. Most recently, studies investigated multiplexed repression with a dCas9-KRAB_ZIM3_-MeCP2 construct, further enhancing inhibition over dCas9-KRAB_ZIM3_ [106]. Subsequent generations of transcriptional repression systems originated through the fusion of dCas-KRAB with direct chromatin modifiers, allowing for long-term gene suppression. For example, the CRISPRoff system developed by Nuñez et al comprises a KRAB-dCas9-DNMT3A-DNMT3L fusion [107]. This work is based on previous dCas systems using DNA methyltransferases such as DNMT1, DNMT3A, and DNMT3B, which can be fused to dCas9 and delivered with the sgRNA of choice. dCas9-induced methylation can effectively reduce expression at the target gene [108–110]. A comprehensive list of such dCas9 fusions can be found in the CRISPRepi atlas developed at the Peking University Third Hospital [111].

Additionally, as with CRISPRa technologies, non-Cas9 cores have been employed for CRISPR-based gene inhibition. dCas12 modalities fused to KRAB provide the benefit of improved multiplexing for CRISPRi [112]. Generating extensive simultaneous targeting with dCas9 would require large designs and multiple promoters to drive each guide [113]. This potential suggests promising applications in diseases with extensive gene dysregulation, such as cancer, and for high-throughput screens (see Section 5.4.1.).

At the mRNA level, dCas13 and its targeting RNA (CRISPR RNA or crRNA) alone can competitively inhibit intracellular RNA binding proteins and block translation [114–117]. In bacteria, the dCas13-crRNA combination achieved higher specificity than dCas9 technologies, but at lower overall inhibition [118]. Apostolopoulos et al corroborated that dCas13 fused to eukaryotic translation initiation factor 4E homologous protein (4EHP) demonstrated high specificity, but lower efficiency than RNAi tools [116]. In line with this specificity, a key benefit of using dCas13 over WT Cas13 is the reduction in collateral activity, wherein Cas13 drives transcriptome-wide destruction after binding to its targets, though this data is limited in mammalian cells [116,115]. Other studies have employed domains that directly modify the mRNA epigenetic landscape, comprising modifications such as removal of methylation of carbon 5 in cytosine (m^5^C), which can be achieved with a TET2 fusion [119]. For therapeutic applications, RNA targeting again allows for controllable, transient reductions in protein production. Additionally, knockdown studies with Cas13 have shown that inhibiting specific RNA molecules in cancer cells can effectively limit tumor growth, emphasizing the relevance of post-transcriptional inhibition driven by dCas systems [120].

Table 2 provides a list of transcriptional repressors and novel enhancements that have been employed in CRISPRi systems.

### 3.3. CRISPRi Therapeutic Applications

CRISPRi boasts significant promise in many disease applications given its versatility and the clinical exploration of its RNAi alternatives. Current *in vivo* applications are discussed below.

#### 3.3.1. Cancer

The variability of cancer phenotypes and frequency of gene or noncoding RNA overexpression greatly support the use of CRISPRi as a therapeutic approach. Mo et al used dCas9-KRAB_ZIM3_ to inhibit the CXC chemokine receptor 1/2 (*CXCR1/2*) gene, a GPCR that has been linked to tumor metastasis when upregulated [126]. Mice were injected with WT cancer cells or CRISPRi targeting *Cxcr1/2* to generate an ovarian cancer model. *In vivo* viability and migration were significantly decreased in mice that received CRISPRi-treated cells [126]. It is important to note that this study did not administer CRISPRi after the tumor had already formed, though.

Li et al investigated a noncoding mRNA-targeted dCas9-KRAB system for the treatment of metastatic breast cancer [127]. Metastatic breast tumors are characterized by abnormal overexpression of microRNA-10b (miR-10b) [128]. Several studies have explored miR-10b inhibition as a method of reducing breast cancer malignancy and proliferation [128–131]. In their study, Li et al delivered dCas9-KRAB and three sgRNAs targeting regions near the miR-10b transcription start site using ultrasound-mediated microbubble destruction (UMMD) technologies. Briefly, the CRISPRi system was housed in a lipid-polymer nanoparticle sensitive to pH changes characteristic of tumor microenvironments, improving site specificity while avoiding viral delivery challenges[127]. Their system significantly decreased miR-10b expression in both mouse and human mammary carcinoma cells. Additionally, in response to this inhibition, levels of downstream miR-10b targets increased. *In vivo*, mouse breast cancer models received CRISPRi or control injections and ultrasound irradiation every 5 days for 20 days post-treatment initiation. Treatment did not significantly decrease tumor weight but decreased the number of metastatic aggregates in the lungs, validating the clinical efficacy of CRISPRi-induced miR-10b inhibition for reducing breast cancer metastasis and malignancy [127].

In enhancing the tunability of CRISPRi for cancer treatment, Gu et al created a dCas9–3xKRAB system, with three fused repressor domains, that could be specifically induced by treatment with oleanolic acid (OA), which has been found to possess tumor suppressive properties [132]. Xenografted mouse models were created using lung and thyroid carcinoma lines treated with control or the inducible CRISPRi system and sgRNAs targeting aurora kinase A (*AURKA*, for the lung cancer model) or *KDM1A* (thyroid cancer model), both of which are associated with increased tumorigenesis. CRISPRi groups subsequently treated with OA displayed the lowest levels of tumor growth and increased expression of pro-apoptotic factors for both cancer models[132]. While, as with Mo et al, CRISPRi delivery was not explored post-tumor initiation, this work of Gu et al provides promise for CRISPRi in treating versatile cancer types with specific, controllable effects.

CRISPRi thus provides a versatile approach to target diverse nucleic acids that contribute to cancer metastasis and severity.

#### 3.3.2. Neurodegenerative Disorders

Like CRISPRa, CRISPRi/CRISPRoff platforms have been employed to target abnormal gene regulation in neurodegenerative diseases.

While Parkinson’s disease is characterized by an extensive, often variable, array of genetic and epigenetic modifications, α-synuclein (*SNCA*) was one of the first to be identified as a dysregulated genetic target. Protein pathology in Parkinson’s is driven by the aggregation of α-synuclein protein. Previous studies with siRNA have demonstrated successful α-synuclein inhibition in non-human primates, and therapeutic effects have been observed with inhibition in human stem cell models [133]. Given the extensive role of α-synuclein in brain function, however, tunable regulation of the *SNCA* gene is preferred to complete silencing or knockdown [134]. Kong et al applied focused ultrasound (FUS) and exosomal vectors to deliver a *SCNA*-targeting dCas9-DNMT3A [134]. dCas9-DNMT3A had previously been targeted successfully to *SNCA* in human induced pluripotent stem cell (hiPSC)-derived dopaminergic neurons obtained from a Parkinson’s patient [135]. The FUS-driven integrated delivery method facilitated *in vivo* adaptations, yielding successful passage of their system, which they refer to as CRISPRi-Exo, across the blood-brain barrier. Functionally, FUS-facilitated CRISPRi-Exo treatment significantly improved motor speed and balance in mouse models of Parkinson’s, while reducing motor delays and pauses. Additionally, treated mice demonstrated lower levels of α-synuclein in the brain, reduced cell apoptosis in Parkinson’s-affected brain regions, and improved neurotransmitter signaling, demonstrating anatomical and clinical benefits [134].

Huntington’s disease involves the accumulation of mutant huntingtin protein (mHTT) in the brain, which exerts multifold effects on metabolic, apoptotic, and neurotransmission processes. However, the WT form, wtHTT, is necessary for these functions, necessitating a method to precisely target the mHTT form. Moreover, studies attempting mHTT knockouts with traditional CRISPR/Cas9 yielded cell death due to the double-stranded DNA breaks. Seo et al delivered a simple dCas9 and mHTT-sgRNA system using a lentiviral vector [136]. The sgRNA was specifically designed to inhibit mHTT more than wtHTT due to the increased number of binding sites in the former. Injection of the lentiviral dCas9-sgRNA system into the striatum of mouse models of Huntington’s disease significantly decreased *mHTT* expression in the striatum with fewer off-target effects and less cell death than Cas9-sgRNA (canonical CRISPR). Additionally, mouse models treated with dCas9-sgRNA showed significant improvements in motor function over untreated or Cas9-sgRNA-treated groups [136]. Again, CRISPRi provides a promising physiological and anatomical therapeutic for neurodegeneration.

Kantor et al further explored CRISPRi usage for Alzheimer’s disease [137]. Their dCas9-KRAB-MeCP2 system was targeted to the apolipoprotein E gene, *ApoE*, the most frequent genetic risk factor for Alzheimer’s. It is especially correlated with late-onset Alzheimer’s disease (LOAD) [138]. This system was injected into the right dorsal hippocampus of mice via their novel AAV-derived vector. Control treatment was injected into the left side. They found that significant inhibition of *ApoE*, up to over 70%, was observed in the right dorsal hippocampus specifically. No significant toxicity was observed, emphasizing the clinical potential. While functional effects on memory or coordination were not observed, their results provide a promising method of delivery and gene repression for Alzheimer’s *in vivo* [137].

#### 3.3.3. Ocular Conditions

Moreno et al initiated an early *in vivo* application of CRISPRi for retinitis pigmentosa (RP), a family of eye disorders involving a gradual loss of photoreceptors in the retina, which is often genetically driven [139,140]. They targeted their dCas9-KRAB system to the *Nrl* gene, which, when inhibited, facilitates the conversion of rod photoreceptors into cone-like cells [140]. This approach helps protect against rod-specific degeneration. Successful *Nrl* repression was confirmed in healthy mice. Then, they delivered the CRISPRi system via a novel, retina-targeting AAV vector injected into the retina of a mouse RP model. CRISPRi treatment significantly improved visual acuity, providing a promising method to tackle visual deterioration due to RP [140]. Burnight et al also applied dCas9-KRAB for RP treatment [141]. Overexpression of the *RHO* gene is a common cause of RP, supporting the use of *RHO*-targeted CRISPRi for treating familial forms of the disease. *RHO* expression significantly decreased in HEK293T cells and an *ex vivo* human retina model. These results were validated *in vivo*. Subretinal injection of an AAV vector with their CRISPRi system into a swine model of photoreceptor degeneration also significantly decreased *RHO* expression in treated eyes up to 12 weeks post-treatment. Importantly, degenerative markers were also significantly lower at both 0 and 12 weeks [141]. Together, these studies demonstrate strong promise for the use of CRISPRi for retinitis pigmentosa, especially given the success in pig models.

Primary open-angle glaucoma (POAG) is another progressive eye disease with several genetic risk factors, including transforming growth factor-β2 (*TGFβ2*) [142,143]. *TGFβ2* has been linked to ocular hypertension, which is highly associated with POAG. In particular, previous studies have demonstrated increased histone acetylation, an activating epigenetic marker, in glaucoma [144]. dCas9-KRAB and an sgRNA targeted to *TGFβ2* decreased *TGFβ2* expression in a human glaucoma cell line when delivered via lentivirus. *In vivo*, mice designed to develop high intraocular pressure were able to retain baseline pressure levels with lentiviral injection of the CRISPRi system and demonstrated decreased ocular inflammation [142]. While promising, clinical implementation of these CRISPRi therapies will depend on precise delivery to the eye to overcome the need for invasive subretinal and intracameral injections.

## 4. CRISPR Epigenetic Regulation in Clinical Trials

In the past two years, the clinical implementations of CRISPR technologies have grown exponentially. Beyond Casgevy, the FDA-approved, *ex vivo* CRISPR/Cas9 treatment available to sickle cell disease and β-thalassemia patients, over 100 clinical trials referencing CRISPR are currently registered with the National Library of Medicine [145]. Additionally, epigenetic CRISPR tools are just starting to enter the clinical trial sphere, rendering an exciting landscape for translational epigenetic therapies.

### 4.1. Tune Therapeutics and Hepatitis B

In November 2024, Tune Therapeutics announced that it would be moving Tune-401, its CRISPRi-based therapy for Hepatitis B (HBV), into Phase 1b clinical trials [146]. Their CRISPRi treatment employs dCas9 and a repressor domain that inhibits via DNA methylation [147]. A corresponding sgRNA targets integrated viral DNA and independent, self-replicating HBV DNA structures known as cccDNA episomes. The CRISPRi system is delivered in mRNA along with the sgRNA in lipid nanoparticles (LNPs), achieving almost complete (99.99%) repression of cccDNA-extracted RNA [148]. These results were corroborated, with observed repression of HBV RNA in HBV-infected mice and of a substitute gene, *PCSK9*, in cynomolgus macaque non-human primates. The latter showed clinically relevant repression for over a year post-treatment, demonstrating the ease with which Tune-401 can enter the medical field [148]. Their study is currently being performed in Hong Kong and New Zealand with adult patients diagnosed with chronic HBV. Participants will receive a single-ascending dose of the LNPs through intravenous administration, and pharmacokinetics, pharmacodynamics, and immunogenicity will be evaluated. It is expected to end by late 2030[149]. Chroma Bio is looking to move into clinical trials in 2025, with a similar, optimized epigenetic approach, CRMA-1001, to HBV that has shown strong preclinical results [150–152].

### 4.2. Epic Bio and Muscular Dystrophy

Epic Bio (Epicrispr Biotechnologies) is another medical company exploring CRISPR-based epigenetic editing. Their novel CRISPRi system, the Gene Expression Modulation System (GEMS), is based on a miniaturized Cas12 protein, CasMINI, developed at Stanford University [153,154]. Currently in clinical trials, Epi-321 is their GEMS-based therapy for facioscapulohumeral muscular dystrophy (FSHD) that functions by regulating *DUX4* expression, the gene responsible FSHD [155]. The nuclease-inactivated miniature Cas protein is fused with repressor domains and delivered via AAV with sgRNA targeted to *DUX4* regulators. Their preclinical results in FSHD patient-derived myoblasts, mice transplanted with FSHD myoblasts, and an FSHD organoid showed significant repression of *DUX4* and significant improvement in functional measures such as cell survival and twitch force [155]. In the clinical trial, which began in May 2025, the EPI-321 AAV is delivered intravenously, with the use of a muscle-specific promoter conferring precise localization [156]. It is currently taking place in the United States (Georgia) and New Zealand with adult type 1 genetic FSHD patients. Two dose levels are being investigated to evaluate safety, transcriptional activity, and *DUX4* expression. It is expected to end in mid-2031 [156].

Tune Therapeutics and Epic Bio’s rise to the clinical trial stage will provide crucial insight into the therapeutic use and efficacy of CRISPR-based epigenetic modifications for diverse diseases. Their results will also strongly encourage the transition of CRISPRa technologies from preclinical to clinical use.

## 5. CRISPRa/i: Current Limitations and Future Outlooks

These diverse disease applications in preclinical *in vivo* models and clinical trials suggest that CRISPRa and CRISPRi will take a strong lead in the next generation of CRISPR therapies. In this section, we discuss outstanding limitations in delivery and safety that must be considered in bringing these tools to patients, then discuss anticipated advancements for the upcoming decade.

### 5.1. Delivery Methods

#### 5.1.1. Viral Vectors

As with other CRISPR therapies, viral vectors are the most commonly used platform for CRISPRa/i delivery in experimental studies, encompassing lentivirus (LV), adenovirus (AdV), and adeno-associated virus (AAV) [157]. LV vectors have a packaging capacity of 8–10 kb, allowing for concurrent delivery of the CRISPR system and sgRNA [157,158]. This larger packaging capacity is especially useful for multipartite systems such as dCas9-KRAB-MeCP2 [159]. Another benefit to LV vectors is their high efficiency and ability to efficiently transduce both dividing and non-dividing cells, such as those in the central nervous system [159,160]. Given the wide applicability of CRISPRa and CRISPRi in the neurodegenerative and neurological disorders discussed in this review, this post-mitotic efficiency is especially beneficial. However, their ability to integrate into the host genome enables long-term expression, which can lead to harmful effects such as mutagenesis and/or tumorigenesis [158,161]. In typical CRISPR/Cas9 applications, this sustained expression can also generate excessive amounts of protein and sgRNA, increasing the risk of off-target edits. While off-target effects pose a lower risk in CRISPRa/CRISPRi due to the reversibility of epigenetic changes, avoiding abnormal gene regulation is crucial for maintaining overall cell homeostasis [2]. Newer third- and fourth-generation LVs contain modifications that increase their safety over earlier versions. Current clinical trials employ third-generation forms due to their self-inactivating ability [162]. Lentiviral vectors are being explored in numerous CRISPR-based clinical trials and approved in five gene therapy products, though all employ *ex vivo* administration [163–166]. Additionally, as of August 2024, ten cloned T-cell products use LV delivery [166]. Thus, lentiviral delivery systems represent a widely used and highly efficient delivery platform for CRISPR epigenetic technologies, especially for *ex vivo* use, but more research is necessary to understand the potential toxicity of sustained CRISPRa and CRISPRi expression.

Adenoviral vectors are substantially larger, with carrying capacities up to 37 kb that permit the delivery of large dCas systems with multiple protein domains. Additionally, they deliver cargo as non-integrating episomes, reducing the risk of mutagenesis and negative off-target effects, which is crucial for precise gene regulation [161]. With better scalability, the key limitation to AdVs is the high immunogenicity of AdV proteins. Additionally, AdVs can regain replicative abilities during production, which can increase cytotoxicity and oncogenicity [167]. Quantification of replication ability and a patient’s immune system strength are thus crucial considerations that must be considered for AdV delivery of CRISPRa or CRISPRi technologies. In fact, only three approved gene therapies are delivered via AdV as of August 2024 [166].

In late 2021, almost half of all viral delivery mechanisms for gene therapies in clinical trials were AAVs, rendering them the most commonly used viral platform in these applications [168,169]. Bearing a packaging capacity less than 5 kb, they are significantly smaller than both LVs and AdVs, which often necessitates co-transduction with distinct particles for the CRISPR system and the sgRNA. Additionally, large multipartite systems cannot be packaged into a single vector, encouraging the use of smaller Cas variants [159]. Alternatively, components of the dCas fusion can be delivered in separate vectors then bind at the target site via split intein-driven trans-splicing [140,170]. This approach has been especially useful in packaging large dCas12-VPR systems for multiplexed activation. Additionally, these “split” dCas systems bring the additional benefit of potential spatiotemporal regulation via chemically-inducible split proteins. Regardless, the benefit of CRISPRa or CRISPRi over canonical CRISPR/Cas9 editing in AAV applications is their lack of need for a template DNA strand that can drastically increase load size [159].

Moreover, despite the potential for low-frequency integration, AAVs typically boast lower immunogenicity and improved safety profiles over LVs and AdVs [169]. AAVs have been successfully and specifically targeted to the lungs and brain in non-human primates, demonstrating their versatility for disease applications [171,172]. These benefits support their use in seven approved gene therapies and numerous CRISPR-based clinical trials [166,173–179]. Moreover, high rates of integration (~47%) are observed at DSBs, which occur during CRISPR/Cas9 editing but not in CRISPR epigenetic regulation, making AAVs even more promising for CRISPRa and CRISPRi [180]. AAV-delivery of a miniature utrophin-targeting CRISPRa for muscular dystrophy successfully increased gene expression without notable side effects in non-human primates [89]. Furthermore, the EPI-321 clinical trial corroborates the clinical relevance of miniaturized dCas systems that allow for efficient delivery of CRISPRi (or CRISPRa) systems using AAV [155].

#### 5.1.2. Nonviral Vectors

Several diverse nonviral vectors are also being explored for gene therapy delivery that can or have been applied to CRISPRa and CRISPRi to protect these systems *in vivo* and avoid many of the risks associated with viral delivery.

Lipid nanoparticles (LNPs) are a common nonviral vector that typically incorporates ionizable cationic phospholipids to create strong interactions with the negatively charged nucleic acid, mRNA in the case of LNPs [181]. The lipid component of LNPs can be modified to adjust safety, scalability, particle size, tissue specificity, and delivery efficiency [181]. Additionally, while the duration of LNP effects is limited by shorter mRNA half-lives, their low immunogenicity enables repeated dosing [182,183]. Low transfection efficiency is an important limitation of widespread LNP use, especially for non-liver tissues, as they tend to accumulate in the liver. For Tune-401, the CRISPRi therapy targeted to hepatocytes, LNPs are especially useful [148]. Recent modifications have also been made to its constituents to promote targeting to bone marrow, placenta, lungs, pancreas, and even the brain [184–188]. While currently only used in one approved gene therapy, LNPs are being explored extensively in CRISPR clinical trials, including Tune-401 [31,149,166,189,190].

Extracellular vesicles provide another nanoscaled, lipid-based delivery mechanism, encompassing exosomes, apoptotic bodies, and microvesicles. Exosomes are the most commonly studied for CRISPR therapies, referring to endocytosis-derived, membrane-enclosed particles with diameters of 30–150 nm [161]. Their natural origin decreases immunogenicity even over LNPs and permits passage through physiological hurdles such as the blood-brain barrier. On the other hand, cellular sourcing also increases variability and makes scalability challenging [191]. While still in the preclinical stage, exosome-based CRISPR delivery has shown promising results *in vivo*, including for Alzheimer’s, osteoarthritis, and hepatocarcinoma [192–194].

Novel synthetic engineered nanomaterials are also being developed for a variety of CRISPR clinical applications. For example, Saha et al investigated modified gold nanoparticles to specifically deliver CRISPR/Cas9 for *in vivo* generation of CAR T cells. Surface engineering modifications were used to minimize systemic degradation and improve bone marrow targeting [195]. To improve the biocompatibility of metal-based systems, Hu et al developed azido hyaluronic acid-based nanoparticles with a copper acetate core for enhanced tumor specificity and cellular uptake. The synergistic effects of their encapsulated CRISPR/Cas9 plasmid and the nanoparticle itself significantly decreased tumor growth and increased apoptosis at the tumor [196]. These novel nanoparticle options provide innovative options for CRISPRa and CRISPRi delivery, especially for cancer, and their adaptability enables additional therapeutic benefits, such as copper-induced tumor cell death and hyaluronic acid-driven targeting of breast cancer cells [196].

Characterizing the delivery of CRISPRa and CRISPRi systems in terms of tissue-specificity and long-term effects will be crucial in progressing them to widespread clinical use. However, promising results and advancements in viral and non-viral CRISPR/Cas9 systems provide a diverse array of platforms that can be adapted for epigenetic use. Key considerations will include the selected Cas protein (ex. MINI versions), number of activator or repressor domains, number of sgRNAs, and target tissue [197].

### 5.2. Off-Target Effects

Off-target binding of Cas9, and thus dCas9, can yield unexpected, potentially harmful effects, including but not limited to indels, oncogenesis, and dysregulation. Early studies suggested that around 3–5 mismatches between the target sequence and sgRNA still permit binding, especially at increasing distances from the PAM site [198]. In a screen of 12 sgRNAs, between 10 and 1300 off-target dCas9 binding sites were observed for a given sgRNA. Importantly, subsequent tests with Cas9 suggested that most of these off-target binding events did not result in DSBs [198]. Other studies reported up to 6000 off-target binding sites for different sgRNAs, most of which occurred in open chromatin, though very few resulted in off-target cleavage [199]. This significant variability between sgRNAs suggests that target regions can be strategically selected to minimize off-target CRISPRa and CRISPRi binding. Moreover, future studies with dCas9-SAM and a non-targeting sgRNA only yielded two off-target binding sites [62]. Additionally, no off-target activation or inhibition was observed with dCas9-VP64 or dCas9 KRAB, respectively, and select sgRNAs [40,42]. Evidently, dCas specificity is highly variable, and though the effects of off-target binding appear minimal, off-target effects must be adequately characterized prior to therapeutic implementation.

To further increase the specificity, precision, and flexibility of CRISPRa/i technologies, many groups have moved beyond optimized guide RNAs and innovative regulatory domains. Inducible systems allow for better control of the CRISPRa/i system and can minimize off-target effects due to long-term expression. Early inducible CRISPRa systems were tested in iPSCs and regulated via small molecules. Several groups have reported on doxycycline (Dox)-inducible dCas9-VPR and SAM systems, demonstrating significant changes in gene expression between Dox addition and removal [54,200–202]. Crucially, Böhm et al demonstrated that while dCas9-VPR expression can be regulated by Dox, an optimal concentration should be identified to maximize efficiency, as higher concentrations do not directly correlate with increased gene expression [203]. Similar doxycycline induction has also facilitated programmable inhibition via dCas9-KRAB, with successful reversibility upon Dox removal [43].

A rapamycin-inducible dCas9 system has also been created by fusing the N- and C-terminal ends of the protein to FRB and FKBP dimerization domains. These domains undergo dimerization in the presence of rapamycin, initiating dCas9 activity [204]. Further spatiotemporal control over such systems is achieved by initially sequestering the two pieces to the nucleus or cytoplasm through the inclusion of nuclear localization or nuclear export signals, respectively [204]. In novel applications, rapamycin induction has been combined with NIR illumination (see below), increasing tissue-specificity and temporal control. Additionally, because rapamycin can cross the blood-brain barrier, this method suggests strong *in vivo* potential for brain-specific CRISPRa/i activation [205].

More recently, Sui et al presented a new drug-inducible CRISPRa/i mechanism based on the human estrogen receptor domain ERT2, which can be activated by tamoxifen or 4-hydroxy-tamoxifen (4OHT) [206]. Specifically, 4OHT facilitated movement of the dCas9 system into the nucleus for transcriptional regulation, with accelerated induction compared to doxycycline systems [206].

Temperature and light induction provide powerful alternatives to these drug-based approaches. For example, Gamboa et al incorporated a “thermal switch” that uses heat shock transcription factor 1 (HSF1) with the transcriptional regulation domain, allowing for increased CRISPRa/i activity with heat treatment *in vivo* and *in vitro* [207]. Heating duration and temperature can further be attenuated to achieve precise levels of dCas expression, providing a way to modulate gene expression levels [207].

UV and NIR wavelengths can also induce split dCas9 systems by stimulating components to bind. NIR is typically preferred due to its reduced toxicity and increased tissue penetration depth [205]. In the visible light region, blue light-inducible or repressible systems have been developed for dCas9, dCas12, and dCas13 [208–210]. Recent iterations have focused similarly on less harmful red and green light regulation of dCas expression [211]. These induction methods are exciting for future therapeutic applications because of the potential for tissue specific, minimally invasive control. Many light-sensitive domains, such as VVD and EL222, are also currently being explored, demonstrating the rise of opto-CRISPRa/i for improving intra- and extracellular specificity [212].

Chemical modifications have also been made to the sgRNA itself. For example, Wang et al describe that sgRNA acylation effectively “masks” its activity, while phosphine-induced Staundinger reduction reactions could subsequently be performed to restore activity in live cells [213]. The versatility of sgRNA modification suggests applicability with varied Cas proteins, effector domains, and even multiplexed systems, but may be limited *in vivo* due to harmful reactants and products.

Incorporating these responsive components allows for increased control over CRISPRa and CRISPRi. Moreover, recent software advancements have permitted the design of precise sgRNAs for use with specific Cas variants. The *crisprVerse* family of R packages analyzes sgRNA for CRISPRa and CRISPRi, accounting for off-target effects as well as epigenetic-specific considerations such as proximity to the promoter [214]. Regardless, thorough off-target testing should be performed at the genome and physiological level for any intended therapeutic application of CRISPRa or CRISPRi technologies.

### 5.3. Immunogenicity

Numerous studies have demonstrated the potential for immunogenic response to the Cas9 protein itself due to its bacterial origins, and nuclease inactivation does not eliminate this risk in dCas9 [215–217]. Yet, many studies in this review did not report significant immune responses to CRISPRa and CRISPRi therapies *in vivo* [76,82,89,134,137]. Moreover, both the Tune-401 and EPI-321 CRISPRi therapies in clinical trials reported strong safety profiles in preclinical NHP studies [148,218].

Off-target effects and immunogenicity are key considerations in continuing to develop CRISPRa and CRISPRi for the clinic. However, predictive technologies, enhanced protein systems, and carefully selected delivery vectors, as well as significant preclinical testing strongly encourage further development.

### 5.4. The Future of CRISPR Epigenetic Editing

#### 5.4.1. Genome-Wide Functional Screens

In this review, we primarily focus on preclinical, therapeutic applications of CRISPRa/i. We would like to note, however, that CRISPRa/i screens are widely used to characterize the relationship between gene expression and phenotypes, particularly in disease [219,220]. Common applications of both systems involve elucidating immune responses, drug resistance, and cancer progression [220,221]. CRISPRa/i can also be used together for comprehensive screens on gene regulation. McCutcheon et al, for example, recently used a pooled CRISPRa and CRISPRi screen to characterize the function of 120 regulators in human CD8+ T cells, identifying BATF3 as an important protein in CAR T cell efficacy for cancer [222]. Schmidt et al similarly used both approaches in T cells to comprehensively identify genes involved in cytokine production [223].

Beyond these specific applications, several multi-center studies are currently employing high-throughput CRISPRa/i screens to characterize hundreds of thousands of pathological genomic changes. The Impact of Genomic Variation on Function (IGVF) Consortium explained that they will use dCas9-based systems to generate perturbations and study regulatory elements and nearby genes in many different cell lines. CRISPRa/i are but one component of the IGVF’s proposal to map and characterize widespread genomic variation [224]. The ENCODE4 Functional Characterization centers have also collected information on the relationship between *cis*-regulatory elements and target gene expression, including via CRISPR screens [225]. Analyses of the centers’ noncoding CRISPR screen datasets investigated crucial factors for these screens, such as the number of sgRNAs necessary to observe significant effects and the optimal number of cells per sgRNA. Ultimately, they found that sgRNAs for CRISPRi ideally occur near distal DNase hypersensitive sites, as they may be located closer to transcription factor binding sites. Regardless, these analyses are primarily focused on K562 cells, a human leukemia cell line, emphasizing the need for broader data sets [225].

Complementary to these analyses, Luo et al utilized a dCas9-KRAB system to determine the function of enhancer-based regulatory networks in human embryonic stem cells. They ultimately found that enhancer function is crucial to cell state transitions, though this impact varies over time [226]. Importantly, CRISPRi allowed them to target a wider range of enhancers than direct deletion, though the inhibition levels achieved by the former are not as strong. Quantitative models further demonstrated that functional enhancers often lie in the same Cohesin and CCCTC-binding factor (CTCF) loop as the promoter [226]. Gschwind et al similarly combined computational models with CRISPR-based dCas9-KRAB to predict, test, and score enhancer-gene interactions. Beyond identifying several disease-specific and cell-specific enhancer mechanisms, they also showed how gene function affects sensitivity to enhancer inhibition, with more extensive expression correlating with lower sensitivity [227]. Together, these studies provide strong mechanisms and potential for the use of CRISPRa/i in comprehensively understanding the human genome in diverse cells and tissues.

#### 5.4.2. Software Technology in CRISPR-based Regulation

Beyond advancements in dCas proteins, sgRNAs, delivery platforms, and induction, machine learning and artificial intelligence also provide promising avenues for enhanced CRISPRa and CRISPRi medicine. AI tools can be used to analyze patient-specific gene patterns, enabling CRISPR-based precision medicine. In relation to the CRISPRa and CRISPRi technologies listed above, AI can provide insight into therapeutic needs based on a patient’s disease profile, including specific genes that are up/downregulated. Potential applications include deriving metabolic pathways for CRISPRa obesity therapeutics; analyzing neural networks for neurodegenerative diseases; and modeling immune system response, pharmacodynamics, and pharmacokinetics for delivery vectors [228]. Additionally, sgRNA selection can be further optimized for minimal off-target binding and enhanced promoter modifications. AI-driven analysis of patient genomics, symptoms, and physiology can also inform drug selection, improving cost-effectiveness for expensive gene therapies [229]. As CRISPR epigenetics advances in the lab, software developments will facilitate scientific understanding and patient use. Key steps in broadening the use of AI to CRISPRa and CRISPRi will be training models with adequate gene regulation data and ensuring patient privacy.

## 6. Discussion

In this review, we provide a thorough summary of the current state of CRISPR-based transcription regulation technologies, namely, CRISPR activation (CRISPRa) and CRISPR interference (CRISPRi), in the clinical realm. With significant leaps in approved CRISPR therapies and clinical trials in the past two years, as well as the two CRISPRi therapies currently in clinical trials, it is a very promising time for the practical development of these tools. Meanwhile, even beyond the clinical realm, the Impact of Genomic Variation on Function (IGVF) Consortium is incorporating dCas-based tools into their functional screens, which will be used to investigate the impact of hundreds of thousands of epigenetic marks [224].

Moreover, with advancements in viral and non-viral delivery vectors to enhance safety and efficiency, physical and software-based methods for precise gene targeting, and AI-driven precision medicine, increasing mechanisms are available to surmount previous hurdles to CRISPRa and CRISPRi boosting the feasibility and translatability of many of the applications for these systems. Thus, we anticipate that CRISPRa/i will continue to play a pivotal role not only in diagnosing but also understanding the breadth of genomic alterations in human disease.

## Figures and Tables

**Table 1.** CRISPR activation systems and structures

Activation System	Reference
dCas9-VP64	Gilbert et al., 2013 [40] Maeder et al., 2013 [41] Perez-Pinera et al., 2014 [42]
dCas9-p65	Gilbert et al., 2013 [40]
dCas9-Rta	Chavez et al., 2015 [54]
dCas9-p300	Hilton et al., 2015 [55]
PRDM9-dCas9	Cano-Rodriguez, 2016 [57]
dCas9-Tet1	Liu et al., 2016 [58]
dCas9-Tet3	Xu et al., 2018 [59]
VP64-dCas9-VP64	Chakraborty et al., 2014 [60]
dCas9-VP64-p65-Rta (VPR)	Chavez et al., 2015 [54]
dCas9-VP64 + MS2-p65-HSF1 (SAM)	Konermann et al., 2014 [62]
dCas9-SunTag-VP64	Tanenbaum et al., 2014 [52]
dCas9-SunTag-p65-HSF1 (SPH)	Zhou et al., 2018 [74]
dCas9 + MS2-MRTFA-STAT1-eNRF2 (DREAM)	Mahata et al., 2023 [75]
dCas12a-VPR	Tak et al., 2017 [65]
dCas12a-p65	Tak et al., 2017 [65]
dCas13b-PABPC1	Torkzaban et al., 2022 [72]
dCas13b–eNAT10	Yu et al., 2025 [71]
dCas13b-ALKBH5	Li et al., 2020 [70]
dRfxCas13d-sgRNA-SINEB2	Cao et al., 2023 [67]
dCas13b-ADAR	Cox et al., 2017 [73]

**Table 2.** CRISPR interference systems and structures.

Repression System	Reference
dCas9-KRAB	Gilbert et al., 2013 [40]
SID4X-dCas9-KRAB	Carleton et al., 2017 [121]
dCas9-DNMT1	Lu et al., 2019 [110]
dCas9-DNMT3A	Vojta et al., 2016 [108] McDonald et al., 2016 [122]
dCas9-DNMT3B	Lin et al., 2018 [109]
dCas9-DNMT3A-DNMT3L	Stepper et al., 2017 [123]
KRAB-dCas9-DNMT3A3L	Nuñez et al., 2021 [107]
dCas9-KRAB-DNMT3A3L	Đorđević et al., 2023 [124]
dCas9-MeCP2	Yeo et al., 2018 [104]
dCas9-KRAB_ZIM3_	Alerasool et al., 2020 [105]
dCas9-KRAB_ZIM3_-MeCP2	Kristof et al., 2025 [106]
dCas12a-KRAB	Ciurkot et al., 2021 [112]
hyperdCas12a-KRAB	Guo et al., 2022 [125]
dPspCas13b	Apostolopoulos et al., 2024 [116]
dPspCas13b-4EHP	Apostolopoulos et al., 2024 [116]
dRfxCas13d-Tet2	Zhang et al., 2024 [119]

## Abbreviations

The following abbreviations are used in this manuscript:

CRISPR: Clustered Regularly Interspaced Short Palindromic Repeats
Cas: CRISPR/CRISPR-associated nuclease
crRNA: CRISPR RNA
tracrRNA: Transactivating crRNA
sgRNA: Single guide RNA (also gRNA)
PAM: Protospacer adjacent motif
DSB: Double-stranded break
SpCas9: Streptococcus pyogenes Cas9
dCas: Nuclease-deactivated Cas
nCas: Nickase Cas
CBE: Cytosine base editor
ABE: Adenine base editor
CPS1: Carbamoyl-phosphate synthetase I
CRISPRa: CRISPR activation
CRISPRi: CRISPR interference
CALD: Cerebral adrenoleukodystrophy
HSPC: Hematopoietic stem and progenitor cell
ABCD1: ATP-binding cassette domain 1
VPR: VP64-p65-Rta
SAM: Synergistic activator mediator
HSF-1: Heat shock protein 1
NAT-10: N-acetyltransferase 10
ALKBH5: m^6^A demethylase AlkB homolog 5
ADAR: Adenosine deaminase acting on RNA
BAT: Brown adipose tissue
WAT: White adipose tissue
Fgf21: Fibroblast growth factor 21
Fndc5: Fibronectin type III domain-containing protein 5
UCP1: Uncoupling protein 1
Sim1: Single-minded family bHLH transcription factor 1
KCC2: K-Cl cotransporter isoform 2
DMD: Duchenne muscular dystrophy
UTRN: Utrophin
MDC1A: Merosin-deficient congenital muscular dystrophy type 1A
ASO: Anti-sense oligonucleotide
RNAi: RNA interference
RISC: RNA-induced silencing complex
siRNA: Small interfering RNA
KRAB: Krüppel-Associated Box proteins
MeCP2: Methyl-CpG binding protein 2
HDAC: Histone deacetylase
4EHP: Eukaryotic translation initiation factor 4E homologous protein
m^5^C: Methylation of carbon 5 in cytosine
CXCR1/2: CXC chemokine receptor 1/2
hiPSC: Human induced pluripotent stem cell
POAG: Primary open-angle glaucoma
TGFβ2: Transforming growth factor-β2
HBV: Hepatitis B virus
LNP: Lipid nanoparticle
FSHD: Facioscapulohumeral muscular dystrophy
LV: Lentivirus
AdV: Adenovirus
AAV: Adeno-associated virus
Dox: Doxycycline
4OHT: 4-hydroxy-tamoxifen
IGVF: Impact of Genomic Variation on Function
